# Balance Recovery Prediction with Multiple Strategies for Standing Humans

**DOI:** 10.1371/journal.pone.0151166

**Published:** 2016-03-14

**Authors:** Zohaib Aftab, Thomas Robert, Pierre-Brice Wieber

**Affiliations:** 1 Faculty of Engineering, University of Central Punjab, Lahore, Pakistan; 2 Université de Lyon, F-69007, Lyon, France; 3 IFSTTAR, TS2, LBMC, UMR_T9406, F-69500, Bron, France; 4 Université Lyon 1, LBMC UMR_T9406, F-69008, Lyon, France; 5 BIPOP team, INRIA Grenoble Rhône-Alpes, LJK Laboratoire Jean Kuntzmann, Univ. Grenoble Alpes, F-38334, Montbonnot, France; Emory University School Of Medicine, UNITED STATES

## Abstract

Human balance recovery from external disturbances is a complex process, and simulating it remains an open challenge. In particular, there still is a need for a comprehensive numerical tool capable of predicting the outcome of a balance perturbation, including in particular the three elementary recovery strategies: ankle, hip and stepping with variable step duration. In order to fill this gap we further developed a previously proposed multiple step balance recovery prediction tool to include the use of the hip strategy and variable step duration. Simulated recovery reactions are compared against observations from different experimental situations from the literature. Reasonable accuracy in terms of step positions and durations were obtained for these different situations using a single set of controller parameters. Moreover, variations in the use of the hip strategy and the step duration between situations were consistent with biomechanical observations. Such a model could be useful to better understand the balance recovery mechanisms, and could also be used to identify potentially hazardous situations.

## Introduction

Falls resulting from loss of balance are a major source of injuries worldwide especially among the elderly. These injuries are a considerable burden on public health-care budgets in many western countries [1, 2] increasing every year due to ageing population. In order to address this problem, it is important to understand the phenomenon of balance recovery and be able to identify potentially hazardous situations. An important step in this regard is predicting the outcome of a balance perturbation and the protective actions taken by humans to avoid a fall.

This issue has been a subject of many experimental research works in the field of biomechanics. Human volunteers are subjected to various balance recovery situations (e.g. slips, trips, pushes, acceleration of the support surface etc.) and their reactions are recorded and analyzed [3–13]. Some fundamental human balance properties have been identified as a result. These include the use of different recovery strategies referred to as ankle, hip and stepping strategies [5, 9, 12], the frequent use of single and multiple steps [8, 9] and the effects of ageing on the recovery ability [6, 7, 13]. Several studies also report thresholds beyond which the balance recovery is not possible. They are expressed in terms of perturbation’s characteristics [4, 6, 12, 13], of the state of the system (position/velocity of the center of mass (CoM)) at the end of the perturbation [10] or at the time of first reaction [11]. However, such thresholds depend directly on the tested situations: disturbance types and characteristics, prior instructions to the subjects, characteristics of the subjects, types of recovery reaction allowed (stepping or not), etc. As such, they cannot be used to predict the outcome of a non-tested condition.

In this context, mathematical modeling offers a complementary way to explore this issue. Several authors focused on predicting the set of states from which it is possible to recover a static balance given the system dynamics, its constraints and the recovery actions allowed. In particular, pioneer works in biomechanics [14] and [15] represented the boundary of the ankle strategy in the instantaneous CoM position-velocity state space. These studies were further expanded to include the hip strategy and a single or multiple recovery steps (see [16] and [17] respectively). As a corollary, this type of modeling can be used to predict the most efficient recovery strategy possible [16–19]. Although interesting, this approach is solely based on the current state of the system and its dynamic properties. It does not explicitly consider the perturbation nor the control aspect of the balance. Such methods are thus not well suited to predict the outcome of a given perturbation, in particular if this perturbation is time varying and the reaction sub-maximal. Moreover, none of the proposed models allow adjusting the step duration, which is either neglected [16, 18], pre-fixed [17] or directly linked to the step length [19], although it is an important parameter of the balance recovery [20].

A second group of modeling schemes focus on balance controllers used in a continuous or intermittent feedback loop with a mechanical representation of the human body [21–28]. An interesting feature of these control schemes is that they are well suited to include the temporal aspects of the balance recovery, in particular the delays and uncertainties in the perception of the system’s state [24, 27]. This approach seems therefore particularly appropriate to model active human reaction in response to external disturbances. However, the current control schemes are limited to quasi-static or slightly perturbed balance where disturbances are compensated by modulation of ankle and possibly hip strategies. None of them include the possibility to step.

Keeping this in view, we previously proposed a balance recovery model which could predict single and multiple stepping responses [29] based on the Model Predictive Control (MPC) approach. This approach is inspired from the prediction capability of human where the balance recovery actions are decided based on the rough estimation of system’s state evolution. However, our earlier model suffered from two major limitations:

1. The human body was approximated using simple inverted pendulum model thus neglecting the role of upper-body inertia (UBI) on balance recovery. Yet it is now well known that humans use this inertia to facilitate balance recovery using the so-called hip strategy described by [5] and others.
2. The step durations were fixed in advance, although it has been observed in human experiments that adjusting the step duration properly is as important as its position [20].

In the current work, we further develop this MPC scheme to include these two properties in our balance recovery prediction tool. The model predictions are then compared against human balance recovery data from the literature. Our aim is to demonstrate the potential and usability of this control scheme to predict human balance recovery behaviors, integrating all three fundamental recovery strategies, including multiple steps if necessary. Such a model could be useful to better understand the balance recovery mechanisms, and could also be used to identify potentially hazardous situations.

## Materials and Methods

### Model Description

**Fig 1:**
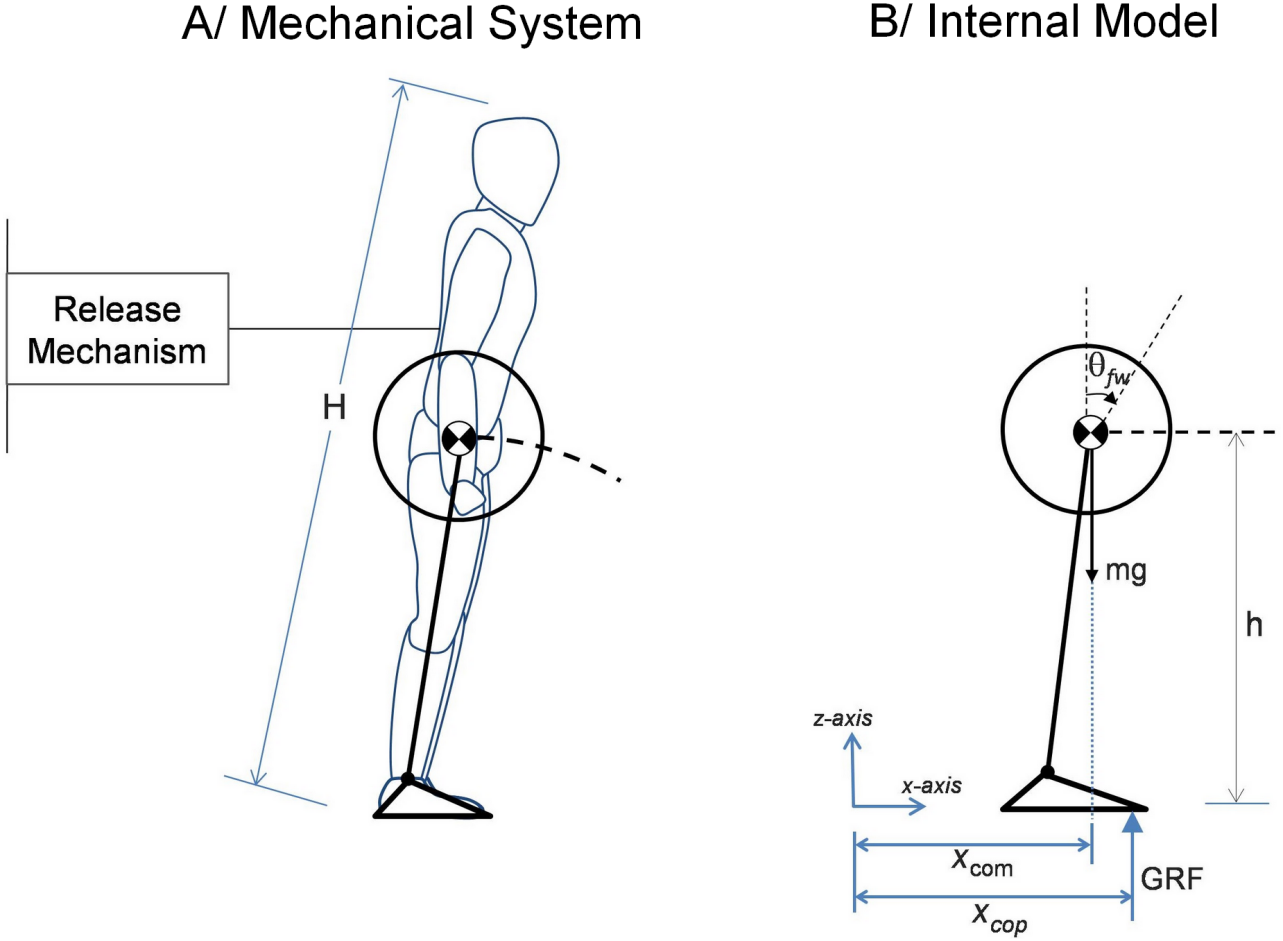
The two representations used of the human body. Left: *Mechanical* system: simple inverted pendulum + flywheel model, i.e. the CoM follows a circular arc. Right: *Internal* model: linearized inverted pendulum + flywheel model, i.e. the CoM travels at a constant height *h*.

We consider human balance recovery in the sagittal plane only. The human body is represented using the well-regarded inverted pendulum model where the whole mass of the system is considered concentrated at the CoM. The model is further supplemented by a foot to represent the base of support [14–17] while a flywheel segment is included to account for the inertial effects of segments’ rotation [16, 17] (Fig 1, left panel). The length of the pendulum is considered constant during each step but can change from one step to another. We call this the *mechanical* system.

Three independent recovery actions are included in this model. They correspond to the mechanical effect of the three classical recovery strategies described in the literature (ankle, hip [5] and recovery step [3]) and also to the 3 control variables of our controller presented in the next section:

- the displacement of center of pressure (CoP) within the foot corresponds to the ankle strategy [5];
- the rotation of the flywheel segment around the CoM corresponds to the hip strategy and arms windmilling [5]. Although it represents more than upper body segments (hip strategy being an antiphase rotation of both the upper and lower part of the body [5]) it is further referred to as the use of the upper-body inertia (UBI) according to Pratt et al. [16].
- the extension of the base of support at the time of step landing corresponds to the stepping strategy [3].

### Principle of Model Predictive Control (MPC)

**Fig 2:**
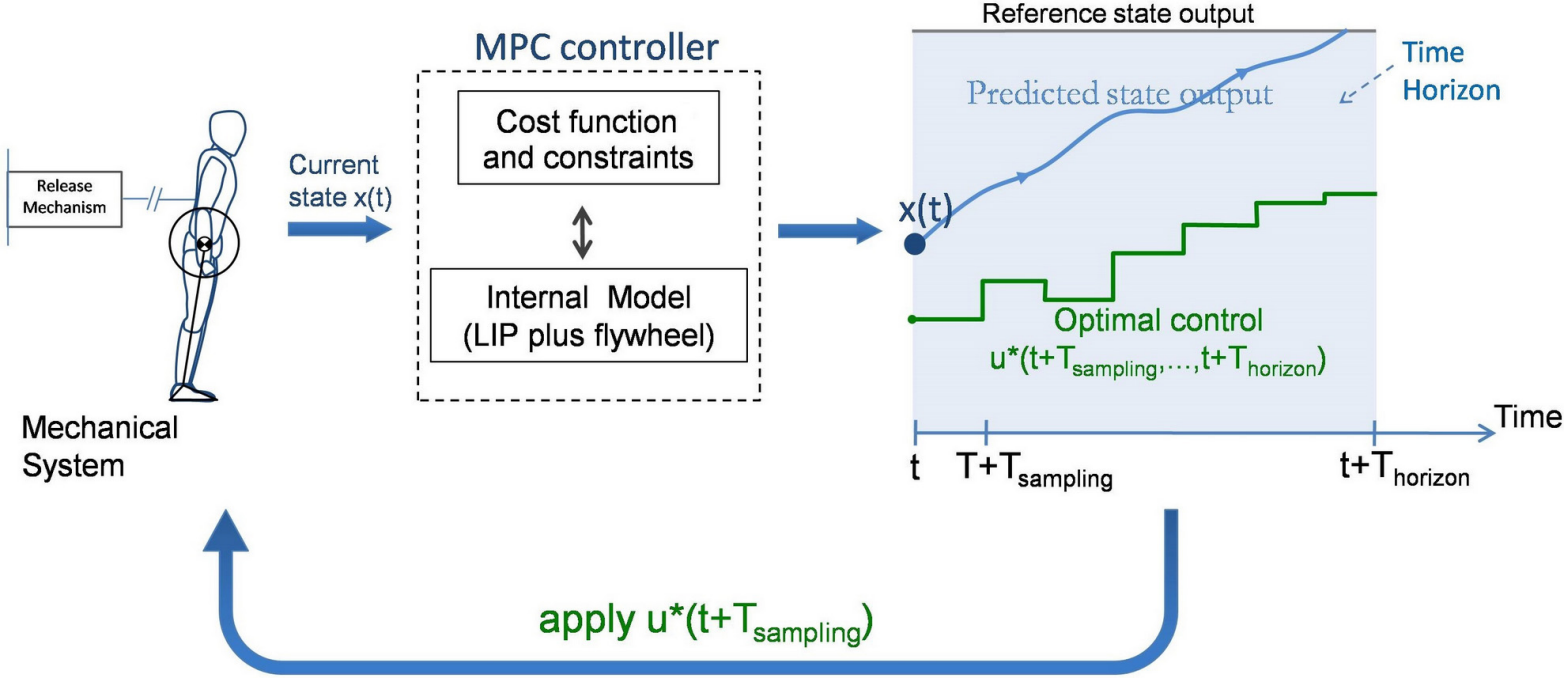
The basic principle of Model Predictive Control. At any time instant *t* the most recent mechanical system state *x*(*t*) is sampled and fed to the controller. The control is computed minimizing the cost function 2 to bring the internal model to a standstill posture.The computed control vector *u*, consisting of the CoM and flywheel jerks and the future step positions, is then applied back to the mechanical system.

This model is placed in closed-loop with a Model Predictive Controller (Fig 2). This type of controller repeatedly solves a series of optimization problems to calculate optimal control strategy based on the simplified model of the system. In our case, it uses a simplified representation of the *mechanical* system, referred to as the *internal* model, to estimate the consequences of the control strategy on future system states.

Each time the controller is called, it chooses the optimal control strategy i.e. the control actions (in our case the position of the center of pressure (CoP), position of the feet and rotation of the inertia wheel) at each time step of the time horizon, that will bring the *internal* model to a desired state. Since the *internal* model is different from the mechanical (or real) system, the control strategy, if applied in its totality to the *mechanical* system, could lead to diverging motions from the expected trajectory. Thus, only the beginning (in our case the first step) of this control is applied to the *mechanical* system and the strategy is updated via a new call to the controller using this most recent system state.

### Internal Model

In our case, the *internal* model is simply a linearized version of the *mechanical* system, which assumes that the CoM moves at a constant height through telescopic legs (see Fig 1, right). The linear dynamics of this system, shown in Eq (1), results in faster and more stable computations:

$${\ddot{c}}_{x}=\frac{g}{h}\left(c_{x}-z_{x}\right)-\frac{j}{mh}\ddot{\theta }$$ (1)

where *g* is the norm of the gravitational force, *h* is the height of the pendulum and *m* is the total mass of the system. *c*_*x*_ and *z*_*x*_ are respectively the x-axis coordinates of the CoM and CoP, $\ddot{\theta }$ is the angular acceleration of the flywheel, while *j* is its moment of inertia.

Though this assumption is not biologically realistic, it has a very limited effect on the final balance recovery behavior. This is due to the fact that only the initial part of the calculated strategy is applied to the mechanical model. The rest is discarded and the strategy is updated as explained earlier.

### Cost Function and Constraints

The optimal control strategy is selected by minimizing a cost function which is primarily defined to quickly bring back the *mechanical* system to a steady state posture. It consists of minilmizing the horizontal CoM velocity and the angular velocity of the flywheel segment over the whole prediction horizon. In addition, we introduce a cost associated to the legswing by minimizing the swing foot acceleration (see discussion about the step duration in the Discussion section). Three additional terms are also included to smooth the motion trajectories and make the CoP converge to under the ankle position at the end of the recovery (see [30, 31]). These latter are only weakly penalized and do not affect much the overall recovery motion. All this results in the following cost function:

$$\min_{u}\;\frac{1}{w_{1}^{2}}∥{\dot{C}}_{k+1}{∥}^{2}+\frac{1}{w_{2}^{2}}∥{\dot{\Theta }}_{k+1}{∥}^{2}+\frac{1}{w_{3}^{2}}∥{\ddot{F}}_{k+1}^{\prime }{∥}^{2}+\frac{1}{w_{4}^{2}}∥{\dddot{C}}_{k}{∥}^{2}+\frac{1}{w_{5}^{2}}∥{\dddot{\Theta }}_{k}{∥}^{2}+\frac{1}{w_{6}^{2}}{∥Z_{k+1}-F_{k+1}∥}^{2}$$ (2)

where ${\dot{C}}_{k+1}$ and ${\dot{\Theta }}_{k+1}$ are the horizontal CoM velocity and angular velocity of the flywheel, ${\ddot{F}}_{k+1}^{\prime }$ the swing foot acceleration, *Z*_*k*+1_ the CoP position, *F*_*k*+1_ the support foot position (projection of the ankle on the ground) and ${\dddot{C}}_{k}$ and ${\dddot{\Theta }}_{k}$ the piecewise constant third derivative of the CoM horizontal position and flywheel angular position. Caps letters stand for column vectors of the values of the variables over the time horizon. Computational details are given in S1 Appendix.

The optimization variables are represented as

$$u={\left[\begin{array}{ccc} {\dddot{C}}_{k} & {\dddot{\Theta }}_{k} & {\bar{F}}_{k+1} \end{array}\right]}^{T}$$

where ${\bar{F}}_{k+1}$ represent the successive step landing position (its relation to *F*_*k*+1_ is described in S1 Appendix). These three variables allow representing the three main recovery strategies: ${\dddot{\Theta }}_{k}$ and ${\bar{F}}_{k+1}$ represent directly the use of upper-body inertia and the stepping strategy respectively, while ${\dddot{C}}_{k}$ ends up controlling the CoP. The terms of the cost function (2) are related to the optimization variables through linear relations detailed in S1 Appendix. As a result, the cost function (2) can be rewritten as a Quadratic Program (QP) under the canonical form:

$$\min_{u}\frac{1}{2}u^{T}Hu+p^{T}u$$ (3)

where *H* is a symmetric matrix and *p* is a column vector.

In addition, the use of the balance recovery strategies is limited (e.g. the CoP has to stay within the boundaries of the foot, the acceleration of the swing foot is limited by physical capacities, etc). The search for the optimal reaction must thus be bounded by kinematic and dynamic constraints. All these constraints, detailed in S2 Appendix, can be written as linear constrained of the optimization variables.

As a result, finding the optimal recovery strategy turns out solving a Quadratic Program (QP) submitted to linear constraints. This can be done very efficiently by a variety of dedicated solvers, such as the quadprog function from Matlab used in this study.

### Timing Issues

Balance recovery reactions are organized in 3 phases:

- An initial reaction time *T*_*reac*_ between the onset of disturbance and the appearance of first mechanical response (controller activation). There is no reaction during this initial phase.
- The step preparation or weight transfer time before the first step initiation *T*_*prep*_. During this phase, the CoP can move and/or the inertia wheel can accelerate, but the first step cannot be initiated.
- The step durations, i.e. duration between consecutive step landings *T*_*step*_ (or between the end of *T*_*prep*_ and the first step landing for the case of the first step).

According to this description, the duration between the onset of the perturbation and the step landings, often referred to as step landing time, is the sum of the three phases *T*_*reac*_, *T*_*prep*_ and *T*_*step*_.

The duration of the first two phases (*T*_*reac*_ and *T*_*prep*_) are considered constant while the step durations *T*_*step*_ are chosen by the controller. However, if this time is left as a free parameter, the optimization problem becomes non-linear, requiring complex numerical solvers. Therefore, for the results of this article, a simple multi-iterative process is employed. At each sample time, multiple simulations are carried out testing different step durations and the corresponding values of the cost function are stored. The optimal step duration *T*_*step*_ is then chosen as the one which results in the minimum value of the cost function.

### Comparison with Experimental Data

In order to compare the predictions of our model with the experimental data, we choose experimental studies involving single and multiple steps under relatively simple perturbations where the subjects are suddenly released from a forward leaning position (tether-release). This is the simplest and hence extensively used experimental protocol in the literature. The subjects fall under the effect of gravity which is already inherently known to them. This circumvents the effect of disturbance perception and anticipation on the balance recovery behavior which are currently not modeled in our system.

Data from the following three studies is used for comparison. These studies are chosen for their thorough description about perturbation and stepping characteristics. They include:

- Single-step recovery results for 4 different inclination levels [6]. Young subjects were inclined forward and asked to recover balance after release by taking a single step no longer than a given length. Inclination was increased until subjects could not recover within this target length. For our comparison, maximal angles, averaged against subjects, were used as input while the step characteristics predicted by the model were compared against the target lengths and step landing times reported in the experiments (step durations *T*_*step*_ were not reported thus comparisons were made on step landing times, i.e. the sum of *T*_*reac*_, *T*_*prep*_ and *T*_*step*_).
- Single-step recovery results for combined inclination and pull force experiments [11]. The subjects were inclined as well as pulled forward at the waist level. Two different combinations of lean angle and pull force is reported, and used as inputs to our models, beyond which the recovery is not possible using a single forward step.
- Multiple-step recovery results [32]. In this case, the number of steps were not constrained. The lean angle was gradually increased until the subject failed to recover balance. The reported average maximum lean angle of 30.7° was used as the input.

### Selection of Model Parameters

The model parameters can be divided into two groups. The first group is related to the experimental scenario under consideration (see the upper half of Table 1). The *mechanical* system is adjusted based on the average reported subject stature and using regressions from [33]. *T*_*reac*_ and *T*_*prep*_ are considered constant according to [3] and [34], while *T*_*step*_ is predicted by the controller among a set of possible values.

**Table 1:** Model Parameters.

Variable	Value
Body height, *H* and mass, *m*	1.63 m and 62 kg for [6]
	1.73 m and 70 kg for [32]
	1.72 m and 67 kg for [11]
CoM height, *h*	0.575 × *H*
Ankle to toe distance, *l*_*Ffront*_	0.123 × *l*_*F*_
Ankle to heel distance, *l*_*Fback*_	0.029 × *l*_*F*_
Reaction Time T_*reac*_	75 ms
Step Preparation Time T_*prep*_	150 ms
Possible step duration T_*step*_	50–500 ms
Horizon length, T_*horizon*_	1 s
Simulation Time	2 s
Sampling Time, T_*sampling*_	25 ms
Max flywheel rotation, *θ*_*max*_	*π*/2 rad
Max flywheel torque, *τ*_*max*_	190 N.m
Flywheel inertia, *j*	8 kg.m^2^
Max swing leg accel., ${\ddot{f}}_{\text{max}}^{\prime }$	180 m.s^−2^
Max ankle-CoM dist., *l*_*max*_	0.85 m
Weight coefficient, *w*_1_	1 m.s^−1^
Weight coefficient, *w*_2_	3 rad.s^−1^
Weight coefficient, *w*_3_	1000 m.s^−2^
Weight coefficient, *w*_4_	100 m.s^−3^
Weight coefficient, *w*_5_	300 rad.s^−3^
Weight coefficient, *w*_6_	30 m

List of model’s parameters and their values used in this study.

The second group of parameters is related to the controller. These parameters were kept constant for all the tested scenarios and are reproduced in the lower half of Table 1. The length of the time horizon (1 s) is chosen so that key balance recovery events, such as steps, appear within this time horizon. The simulation time is chosen sufficient enough (2 s) to allow complete recovery. The sampling time (25 ms) is chosen small enough to allow quasi-continuous control.

An important ingredient in the proposed controller is the choice of the different weights in the cost function (2). Since the basic objective of the controller is to reach a standstill posture after a perturbation of varying magnitude, the major recovery criterion is the CoM velocity, whose weight is chosen by default, *w*_1_ = 1 m.s^−1^. In this respect, the last three terms, penalizing the motion jerks and CoP centering, are introduced only to get desirable motion characteristics of low importance, i.e. smoothness and a comfortable final posture, without affecting the step length(s) and duration(s) during the recovery behavior. Indeed, their absence results in undesirable oscillations, as demonstrated in a previous work [31]. Typical values of *w*_4_ and *w*_5_ between 100–1000 m.s^−3^ and rad.s^−3^ respectively, and *w*_6_ between 10–100 m are found to be appropriate for this simple purpose. More important are the weights *w*_2_ and *w*_3_. The first one has to be chosen in order to obtain a realistic scaling of the reaction of the upper-body inertia with the perturbation level, as observed by [27], and a range of 3–30 rad.s^−1^ is proposed for this. The value of *w*_3_ is chosen after observing the relative order of magnitude of the swing foot acceleration cost with respect to the initial CoM velocity cost. For a unit value of *w*_1_, the value of *w*_3_ is of the order of 100 m.s^−2^ [31]. A higher value is proposed in Table 1, so a weaker penalization, to ensure that the reduction of the CoM velocity remains the main objective in our cost function. How the different motion recovery behaviors are affected by changes in these parameters is analyzed more precisely in the subsection Sensitivity Analysis below, leading to simple guidelines for selecting them.

The constraint parameters related to the different recovery strategies (ankle, hip and stepping) are chosen to match the capabilities of an average human. The size of the foot, which limits the amplitude of the ankle strategy, is calculated from subjects’ average stature using regressions from [33]. Flywheel torque and moment of inertia values are taken from [35]. Step size is limited by both the peak foot acceleration and the maximum distance between support foot and CoM, whose values are chosen after observing real human subject data from [36]. All these constraints can be written as linear functions of the optimization variables which are developed in details in S2 Appendix.

## Results

### Comparison between experimental and simulated results

**Fig 3:**
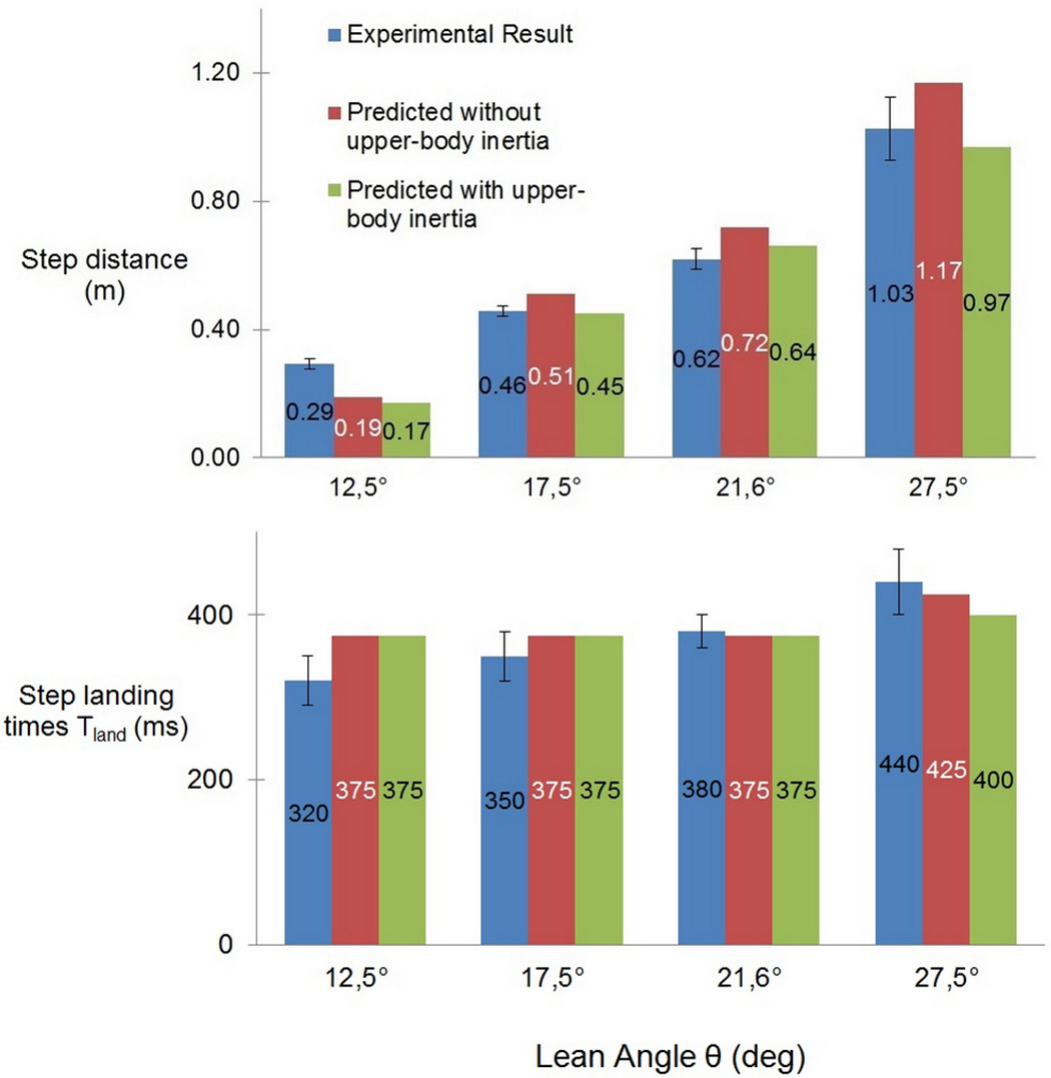
Step lengths and landing times for single step recovery scenarii from [6]. Experimental (blue bars, averaged across subjects ± one standard deviation) versus simulated results, without (red) and with (green) the consideration of upper-body inertia.

**Fig 4:**
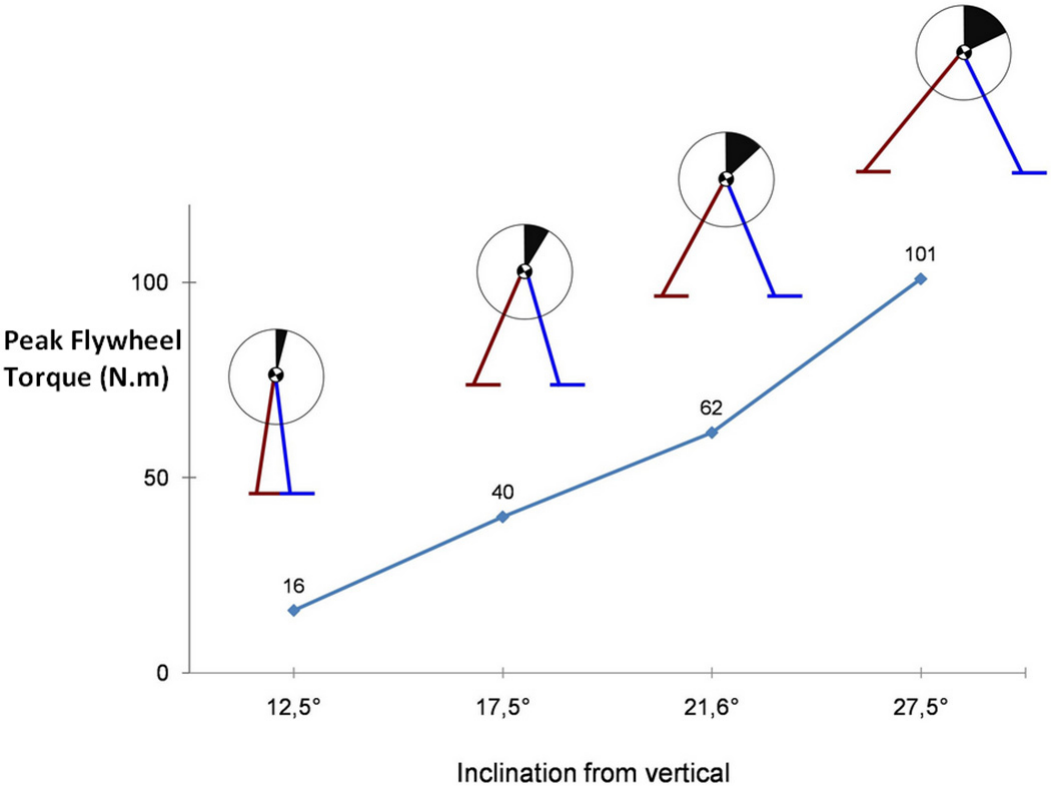
Peak of flywheel torque achieved during the recovery scenarii from [6] and corresponding recovery postures at the instant of stepping. The filled area represents the flywheel rotation angle.

**Fig 5:**
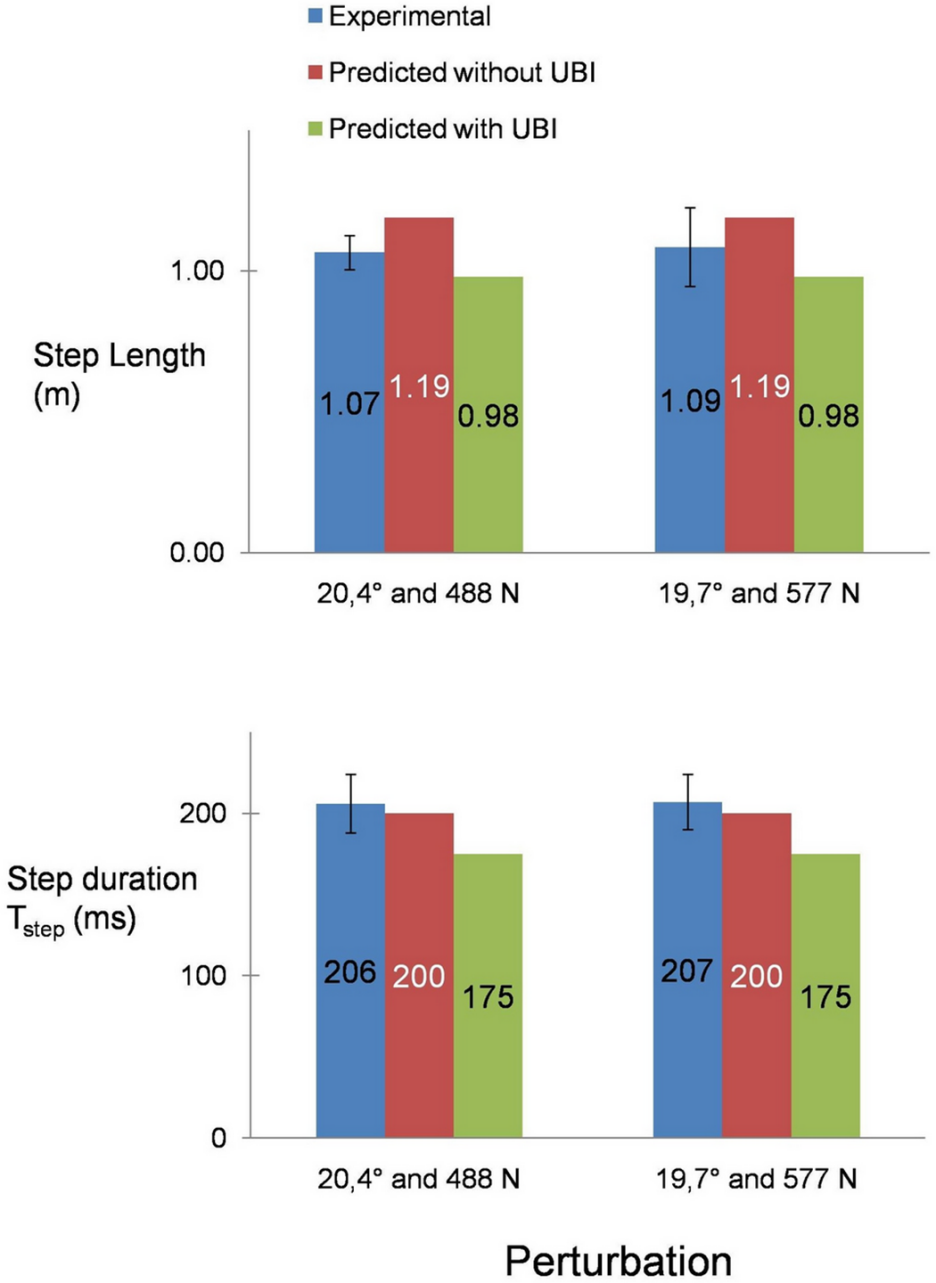
The single-step recovery predictions for 2 scenarii from [11] with and without upper-body inertia against the experimental results.

**Fig 6:**
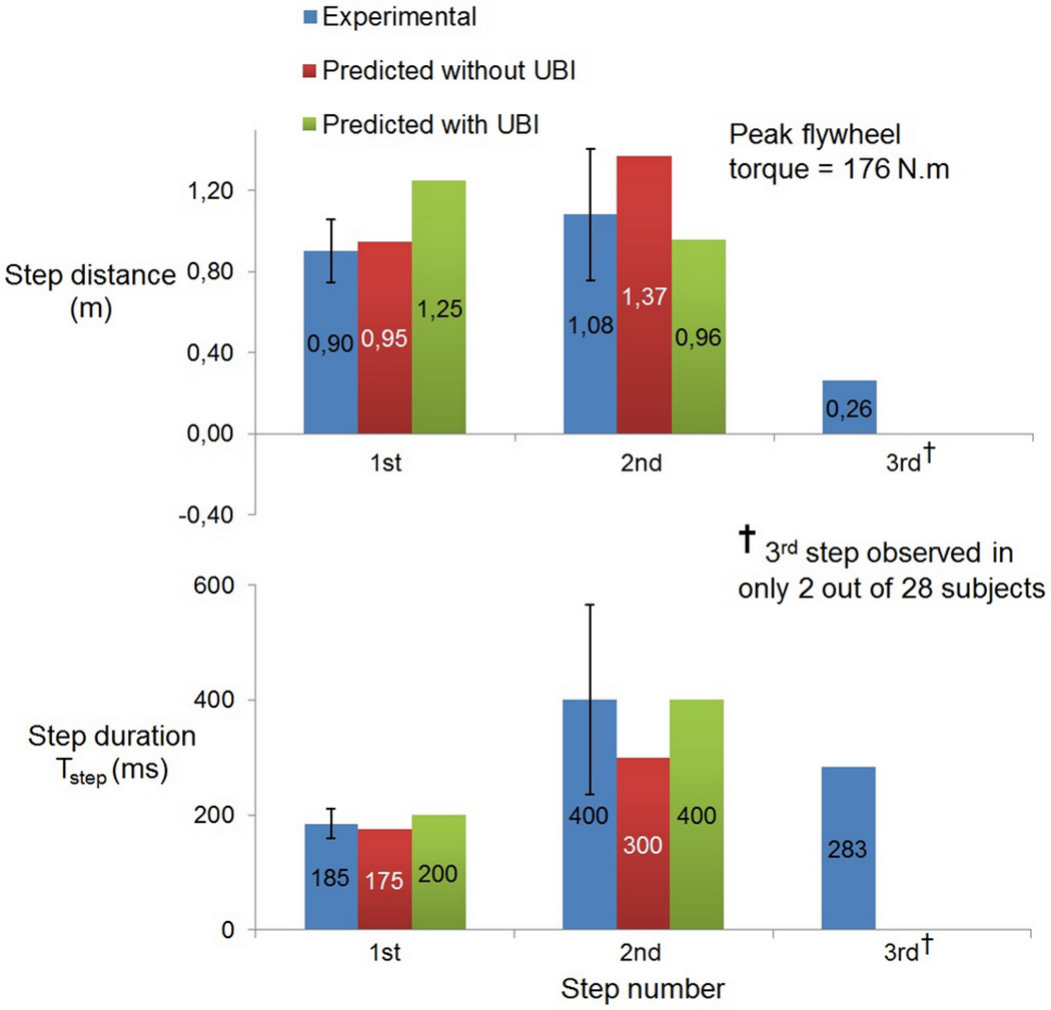
Stride length results for multiple-step recovery scenario from [32]. The 3^*rd*^ stride, reported by [32] but not predicted by our model, was only observed for 2 out of 28 subjects.

Comparison between the experimental and simulated results is shown in Figs 3 to 6. Blue plots show the experimental results (averaged across subjects ± s.d.). In order to explicitly observe the effect of the use of upper-body inertia, simulation results are plotted separately in the absence (red plots) and presence (green plots) of the flywheel segment.

Fig 3 shows optimized step length and step landing times (sum of *T*_*reac*_, *T*_*prep*_ and *T*_*step*_) for single-step recovery in different release angles [6]. It can be perceived that as a whole the results of our controller are in agreement with the experimental data both in terms of step lengths and landing times. The introduction of upper-body inertial effects in the model reduces the recovery step length by about 8–17%. The use of ankle strategy (not shown) is generally unaffected due to a weak *w*_6_ penalization leading to its maximal use throughout the motion.

Fig 4 shows the corresponding peak torque and rotation angle of the flywheel used by our controller for each inclination. It can be seen that the use of upper-body inertia scales well with the inclination level.

Fig 5 shows the results for the single-step recovery scenario where the subjects are inclined and pulled forward at the waist level [11]. Being at the single-step recovery limit, almost identical step lengths and durations are reported in the study for both perturbations, which match well with our predicted results.

Lastly, Fig 6 shows the stepping results for the multiple-step recovery scenario [32]. Again, the results match well with the experimental data. However, the use of upper-body inertia combines with a larger 1^*st*^ stride duration (200 ms), leading to even larger stride length. Note that the 3^*rd*^ stride, reported by [32] but not predicted by our model, was only observed for 2 out of 28 subjects.

### Sensitivity Analysis

By construction, varying *w*_6_ or *w*_1_, as long as *w*_1_ remains small compared to the other weight coefficient (i.e. the priority is given to zeroing the CoM’s velocity), has a very small effect on the balance recovery behavior. This point was already shown in one of our previous study [29].

**Fig 7:**
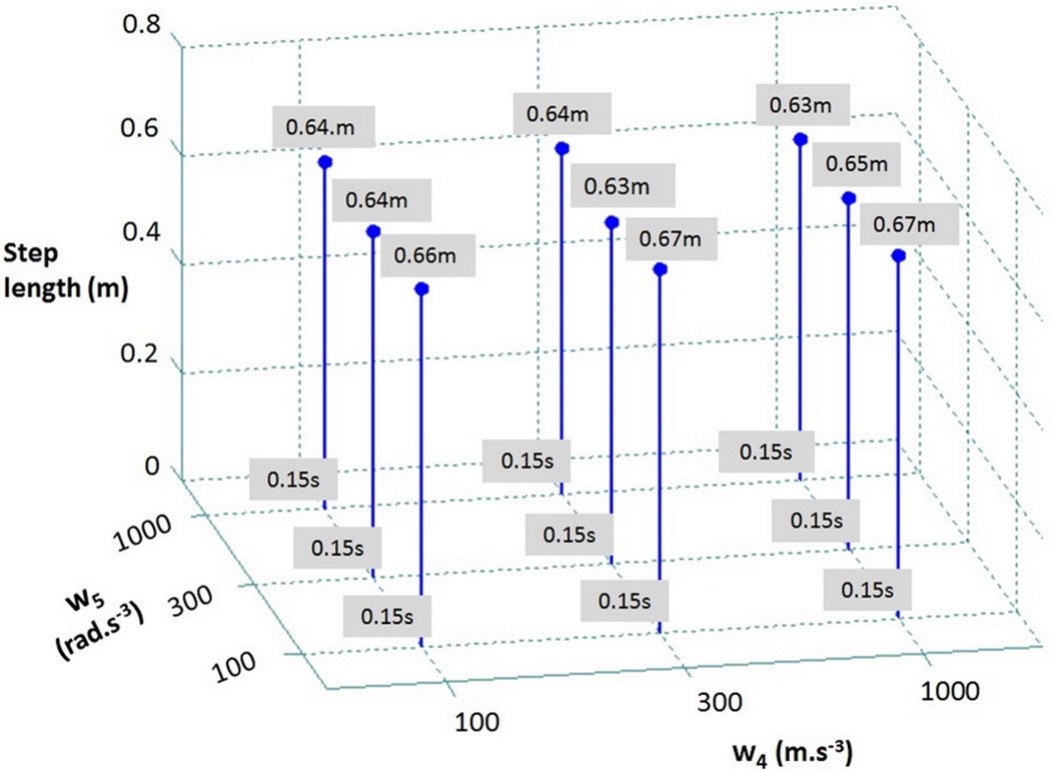
Effect of varying weight coefficients w_4_ and w_5_ on the resulting predicted single step length and duration for the inclination of 21.6° from vertical.

In order to show the effect of varying the other weight coefficient values, we chose a single step scenario from [6] where the subjects are released from 21.6° from vertical. The effect of varying weight coefficients *w*_4_ and *w*_5_, which penalize the CoM and flywheel jerks respectively, on the predicted step length and duration is shown in Fig 7. All other parameters are set according to Table 1. It can be seen that their effect is small (around 6%) on the step length and no effect on the step duration.

**Fig 8:**
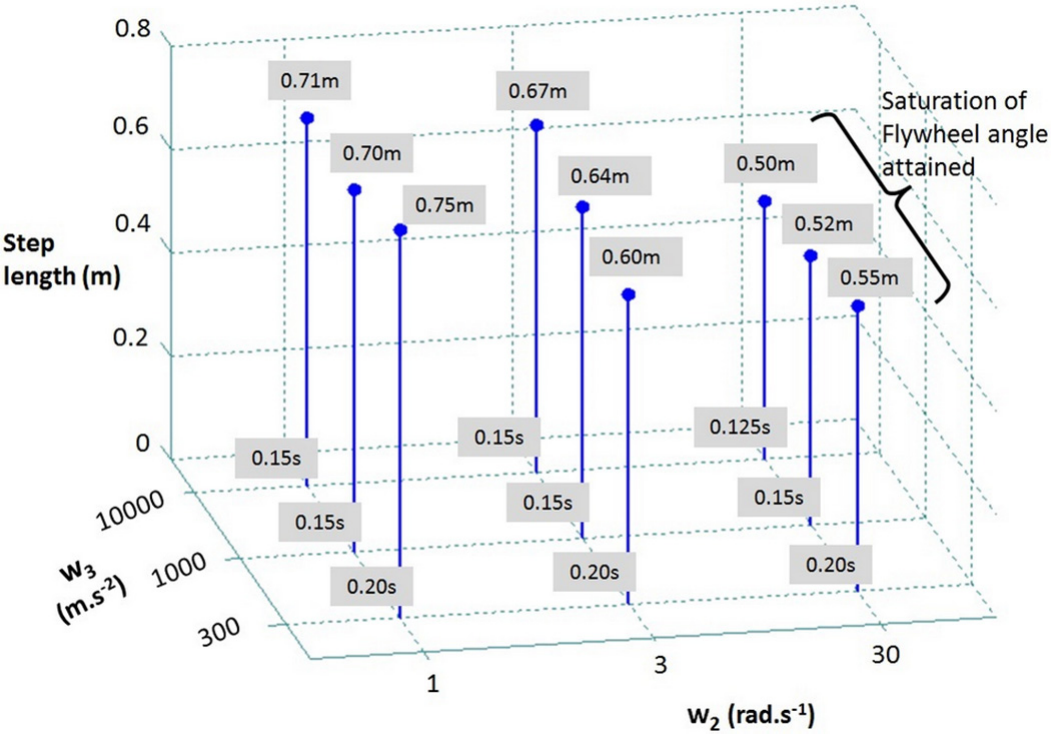
Effect of varying weight coefficients w_2_ and w_3_ on the resulting predicted single step length and duration for the inclination of 21.6° from vertical.

Fig 8 shows a similar comparison for the weight coefficients *w*_2_ and *w*_3_. The increase in the value of *w*_2_ from left to right (leading to smaller penalization of flywheel) systematically reduces the step length due to an increase in the upper-body rotation. The weight coefficient *w*_3_ also has a noticeable effect on the stepping characteristics. As its value is decreased (leading to stronger penalization of the swing foot acceleration) the optimal step duration tends to increase.

These variations of the different balance recovery strategies with respect to different choices of weights look reasonable and as expected, suggesting a straightforward and natural procedure for selecting these weights. We believe this is an advantageous aspect of the Model Predictive Control that we propose.

## Discussion

The presented balance recovery scheme shows a great potential of generating a human-like recovery response to external balance perturbations. In particular, we demonstrated that, using a single set of parameters and anthropomorphic constraints, the model reproduced the balance recovery characteristics observed for different perturbations types (lean-and-release with or without pulling force) and different perturbation levels. Such model would be useful not only to predict the reaction of subjects in different situations but also to gain a better understanding of the balance recovery mechanisms (e.g. quantification of the effects of the different balance recovery mechanisms or of the anthropomorphic constraints).

### Hip Strategy Modeling

Hip strategy is usually defined by its kinematic characteristics, i.e. the antiphase rotation of both the upper and lower part of the body. However, from the original paper from Horak and Nashner [5], the hip strategy can also be defined by its mechanical effect, i.e. a horizontal shear force under the feet that tend to decelerate the CoM. A similar mechanical effect can also be produced by windmilling the arms. The resulting mechanical effect was modeled through a simple flywheel centered at the CoM of the system and we referred to it as the use of the upper-body inertia (UBI), as already proposed by Pratt et al. [16]. This choice results in a linear model leading to fast and robust computations. Note however that the flywheel should not be considered as a representation of the trunk: bending the trunk forward has a geometrical effect on the CoM (it moves it forward) while slowly rotating the flywheel has not.

Using this flywheel, i.e. the hip strategy, our controller tends to limit the recovery step length (compare red and green bars in upper panels of Figs 3 and 5) and results in a closer approximation of the experimental step lengths. The ankle strategy is always put to its maximum due to small penalization of the CoP divergence from the ankle position (weight coefficient *w*_6_). Moreover, it can be observed in Fig 4 that the use of UBI scales with the perturbation level, both in terms of peak torque and rotation angle. This is consistent with the biomechanical observations [26], which support our modeling choice.

In some cases, the stepping predictions without the flywheel segment compare better with the experimental data (e.g. Fig 6, 1st step) which might question the usefulness of including this segment in our model. Nevertheless, we have observed that the UBI has a non-negligible effect on recovery step [37] and should be included for a complete balance recovery model. In the absence of any experimental data on the actual usage of UBI in balance recovery, we speculate that the apparent inaccuracy may result from to the way constraints are defined in our model. The exploitation of a particular strategy by humans (especially the hip strategy) is somehow related to what extent other strategies are put in place. For example, in reality, taking a large step while simultaneously accelerating the upper-body to its limits is extremely difficult, if not impossible. This is due to the varying torque-producing capacity of the hip muscles at different angles. This could not be represented with the current optimization scheme as it only considers constant constraints and control parameters. One way to represent this phenomenon is to link the constraints and/or the control parameters to kinematic parameters though non-linear relationships. This, however, would require a more complex / less stable optimization solver.

### Step Duration as a Free Parameter

A major contribution of this article is the optimization of step durations. It has been observed in human experiments that, following a balance perturbation adjusting the step duration properly is as important as its position [20]. However, in most of the previous models involving recovery stepping, the step duration is either neglected (instantaneous stepping as in [18]) or is considered known and pre-fixed (e.g. [17, 29]) to simplify the model. Indeed, including step duration as an optimization variable renders the optimization scheme non-linear, requiring specialized non-linear MPC solvers [38]. As this study did not focus on real-time control of bipeds, this issue was handled in a simpler way: the overall non-linear problem was replaced by a series of simple QP for which the step duration were prefixed.

A previous attempt to adjust the step duration [38] ended up in optimized steps durations always as quick as possible. One of the probable reasons is that, by assuming mass-less legs, mechanical and energetic cost associated with leg movement [39, 40] were neglected. To counter this, we introduce here a simple model of this mechanical cost in the objective function by penalizing the forward acceleration ${\ddot{F}}_{k+1}^{\prime }$ of the swing foot. We showed that it leads to a non-monotonous cost function regarding the step duration, i.e. a local minimum appears for a non-zero step duration [41].

As a result, the simulated stepping duration varies within its possible range between the different recovery situations (between 150 ms to 400 ms, see Fig 3 and second stride of Fig 6). In particular, step durations tend to slightly increase with the perturbation level (see Fig 3). Although this increase remains small compared to the experimental results, the tendency is still in the right direction. Further experimental data, in particular about the kinematics of the swing foot for different perturbations, would be required to further improve the current model.

### Possibility of non-stepping responses

Though the current article intends to compare the recovery responses involving steps, it is worth noting that this model is also capable of reproducing such responses for smaller perturbations. This behavior is shown in our earlier publications [31, 41] and are briefly discussed here. For very small perturbations, the model chooses solely the ankle strategy with negligible use of upper-body inertia and a negligible step length. As the perturbation level is gradually increased, the hip and stepping strategies start to appear. However, both these strategies appear before ankle strategy limit is reached. Qualitatively, these results go well with the experimental biomechanical data. The co-existence of ankle and hip strategies before reaching the ankle strategy limit has been reported in the biomechanics literature when stepping is disallowed (e.g. [26]). Moreover, it was shown in the experiments that when stepping is allowed, it is also initiated well before the stability limit of the so-called “fixed support strategies” is reached [8, 9]. However, in these studies, no data is reported on the actual level of usage of each strategy by the subjects. This severely limits our ability for a quantitative comparison and validation of our model for non-stepping responses.

### Future Works

Although the current results are encouraging, the proposed balance recovery tool still needs to be further developed and tested. For example, the model should be extended to examine the response of elderly population which are particularly at high risk of falling. This requires the modification of relevant age-related parameters in the model such as sensorimotor and muscular decline. In preliminary studies [31, 42] we demonstrated that, by only adjusting constraints (increasing *T*_*reac*_ and *T*_*prep*_ and reducing ${\ddot{f}}_{\text{max}}^{\prime }$) this model could also correctly reproduce results from [6] regarding elderly subjects. Further developments are underway to refine this model.

Similarly, performances of the model should be tested in more complex situations of perturbation, typically time varying, long lasting perturbations. In such situations, it is likely that sensory and cognitive aspects (e.g. sensory delay, noise and integration, inclusion of the estimated perturbation in the reaction, etc.) will have to be considered and included in the model. This would also pave the way for representing the behavior of different types of populations such as the elderly. Non maximal recovery performance should also be looked at, possibly through an adjustment of the control weight parameters (see preliminary works on this topic in [31]).

## Supporting Information

S1 Appendix Relation between variables of the cost function. (PDF)

S2 Appendix Kinematics and dynamics constraints. (PDF)
