# Supplementary material for: Balance Recovery Prediction with Multiple Strategies for Standing Humans
Source: PLoS One. 2016 Mar 14;11(3):e0151166. doi: 10.1371/journal.pone.0151166 (PMC4790971; doi:10.1371/journal.pone.0151166)
Supplement: S1 Appendix — (PDF) [file pone.0151166.s001.pdf]

Appendix S1: Relation between variables of the cost function

Variables represented with capital letters stand for column vectors of values over the prediction horizon, e.g.

$$C_{k+1} = \begin{bmatrix} c_{k+1} \\ \vdots \\ c_{k+N} \end{bmatrix} \tag{S1.1}$$

Time derivatives

The position, speed and acceleration of the CoM over the whole prediction horizon can be related to the CoM state of the system at a time t_k, $\hat{c}_k = \begin{bmatrix} c_k & \dot{c}_k & \ddot{c}_k \end{bmatrix}^T$, and the piecewise constant third derivative $\dddot{C}_k = [\dddot{c}_k, \dots, \dddot{c}_{k+N-1}]^T$ through constant matrices:

$$C_{k+1} = S_p \hat{c}_k + U_p \dddot{C}_k, \tag{S1.2}$$

$$\dot{C}_{k+1} = S_v \hat{c}_k + U_v \dddot{C}_k, \tag{S1.3}$$

$$\ddot{C}_{k+1} = S_a \hat{c}_k + U_a \dddot{C}_k. \tag{S1.4}$$

Details of these matrices can be found in [30]. Identical relationships can be derived for the motion of the flywheel segment.

Center of Pressure

The linear dynamics (1) can be reversed to compute the position of the CoP as:

$$z_x = c_x - \frac{h}{g}\,\ddot{c}_x - \frac{j}{mg}\,\ddot{\theta} \tag{S1.5}$$

The position of the CoP over the whole prediction horizon Z_{k+1} can then be related to the piece-wise constant third derivatives $\dddot{C}_k$ and $\dddot{\Theta}_k$:

$$Z_{k+1} = S_z \begin{bmatrix} \hat{c}_k \\ \hat{\theta}_k \end{bmatrix} + U_z \begin{bmatrix} \dddot{C}_k \\ \dddot{\Theta}_k \end{bmatrix}, \tag{S1.6}$$

with

$$S_z = \begin{bmatrix} S_p - \frac{h}{g} S_a & -\frac{j}{mg} S_a \end{bmatrix}, \tag{S1.7}$$

$$U_z = \begin{bmatrix} U_p - \frac{h}{g} U_a & -\frac{j}{mg} U_a \end{bmatrix}. \tag{S1.8}$$

Foot position

The position of the support foot over the whole prediction horizon F_{k+1} can be related to the current support foot position f_k, which is fixed on the ground, and the positions $\bar{F}_{k+1}$ of the future steps, which is an optimization variable. If the step durations are already known, this can be done easily with matrices V_{k+1} and $\bar{V}_{k+1}$ filled with 0s and 1s simply indicating which sampling times t_i fall within which steps:

$$F_{k+1} = V_{k+1} f_k + \bar{V}_{k+1} \bar{F}_{k+1} \tag{S1.9}$$

Swing foot acceleration

The motion of the swing foot is interpolated in the forward/backward direction with 5th degree polynomials between its current position, velocity and acceleration and the future positions of the foot on the ground, with zero velocity and acceleration (no impact). This trajectory is further discretized. Acceleration of the swing foot $\ddot{f}'_j$ at each of these discretization instants t_j can then be related to the step landing position via a linear relation:

$$\ddot{f}'_j = a_j \bar{F}_{k+1} + b_j \tag{S1.10}$$

where a_j and b_j only depend on the discretization instant t_j and the current state of the swing foot. This relation can be extended over the whole prediction horizon:

$$\ddot{F}'_{k+1} = A\bar{F}_{k+1} + B \tag{S1.11}$$

where A is a matrix whose rows correspond to discretization times t_j and columns to steps, and B is a column vectors.
Note that the foot is assumed to be sufficiently removed from the ground during its motion and its effects on the system dynamics are neglected (LIP assumption).
