# Supplementary material for: Balance Recovery Prediction with Multiple Strategies for Standing Humans
Source: PLoS One. 2016 Mar 14;11(3):e0151166. doi: 10.1371/journal.pone.0151166 (PMC4790971; doi:10.1371/journal.pone.0151166)
Supplement: S2 Appendix — (PDF) [file pone.0151166.s002.pdf]

Appendix S2: Kinematics and dynamics constraints

The balance recovery is subjected to a series of kinematic and dynamic constraints that have to be satisfied over the whole prediction horizon, for all $i \in [k+1, \ldots, k+N]$. These include:

1. Direct linear bounds on the flywheel rotation in terms of its angle and torque

$$\|\theta_i\| \leq \theta_{max} \tag{S2.1}$$

   and

$$\|j\ddot{\theta}_i\| \leq \tau_{max} \tag{S2.2}$$

   where the rotation angle θ_i and the acceleration $\ddot{\theta}_i$ at each instant are related to the optimization variable $\ddot{\Theta}_k$ through recursive relations of the form (S1.2) and (S1.4).

2. Upper bound for the extension of the support leg, enforced by limiting the distance between the horizontal position of the CoM and the horizontal position of the support foot on the ground:

$$\|c_i - f_i\| \leq l_{max} \tag{S2.3}$$

   with c_i and f_i related to the optimization variables $\dddot{C}_k$ and $\bar{F}_{k+1}$ through equations (S1.4) and (S1.9) respectively.

3. Upper bound for the acceleration of the swing foot:

$$\|\ddot{f}'_i\| \leq \ddot{f}'_{max} \tag{S2.4}$$

   where $\ddot{f}'_i$ can be related to the optimization variable through equation (S1.11). This constraint limits the step length for a given step duration.

4. Constraints on the CoP, which has to stay within the boundaries of the foot:

$$D(z_i - f_i) \leq b, \tag{S2.5}$$

   where D and b are a matrix and a vector encoding the shape of the foot with respect to the position f_i of the support foot on the ground. In our 2D case, with instants t_i falling always during single support phases, the relation D is reduced to two simple linear bounds:

$$\begin{cases} (z_i - f_i) & \leq l_{Ffront} \\ -(z_i - f_i) & \leq l_{Fback} \end{cases} \tag{S2.6}$$

   Again z_i and f_i can be related to the optimization variable through equations (S1.6) and (S1.9) respectively.

All these constraints can be expressed as a set of linear inequality constraints on the optimization variables.
